# A national survey on registered products, availability, prices, and affordability of 100 essential medicines in community pharmacies across Sri Lanka

**DOI:** 10.1186/s12913-023-10137-y

**Published:** 2023-10-19

**Authors:** Chiranthi Kongala Liyanage, Mekala Gunawardane, Pamodee Panchalee Kumaradasa, Priyanga Ranasinghe, Raveendra Laal Jayakody, Priyadarshani Galappatthy

**Affiliations:** Department of Pharmacology, Faculty of Medicine, University of Colombo, 25 Kynsey Rd, Colombo, 00800 Sri Lanka

**Keywords:** Affordability, Availability, Community pharmacies, Essential medicines

## Abstract

**Introduction:**

Availability of essential medicines that meet the expected quality standards, in appropriate dosage forms at affordable prices is a fundamental prerequisite to fulfill healthcare needs of given a population. This study assessed available products, prices and affordability of essential medicines (EM) in community pharmacies in Sri Lanka with comparison of registration status from the National Medicines Regulatory Authority(NMRA).

**Methods:**

A cross-sectional island-wide survey of 80 pharmacies was conducted according to World Health Organization and Health Action International Manual (WHO/HAI). Hundred medicines were selected from the global core list(n = 14), regional core list(n = 16) and the Sri Lanka Essential Medicine List (SL-EML) (n = 70) based on healthcare needs. Number of registered products in 2015 and 2021 were compared.

**Findings:**

Average availability was 85.4%(± 12.31) and availability was lowest in the Northern province (69.38 ± 21.18%)(p = 0.008). Availability between the state owned, franchise and privately owned pharmacies was not significantly different (p > 0.05). 89.4% medicines were affordable except for amiodarone, hydroxychloroquine, sitagliptin, soluble insulin, isophane insulin, losartan, levodopa carbidopa combination, clonazepam and ceftriaxone. The median price ratio (MPR) of 33.7% of medicines was less than 1 and MPR of 37.1% originator brands (OB) was over 3. Median number of generic brands in the market was 8(range 2–44), 9% of medicines had 20 or more products in the market and 72.7% medicines had more products available than the number registered in 2015. The average number of registered products were similar in 2015 (8.27) and 2021(7.59) (p = 0.15).

**Conclusion:**

The overall availability of EMs in Sri Lanka was high in all categories of community pharmacies. Medicines were largely affordable and reasonably priced in 2015, although OBs were generally more expensive. Majority of medicines had more products in the market than the number of registered products.

**Supplementary Information:**

The online version contains supplementary material available at 10.1186/s12913-023-10137-y.

## Introduction

Essential medicines (EM) are defined by the World Health Organisation (WHO) as medicines that satisfy the priority health needs of a population. They are chosen based on evidence of efficacy, safety, and cost-effectiveness as well as disease burden in a country or a region [1]. Therefore, availability of EMs that meet the expected quality standards, in adequate quantities, in appropriate dosage forms, at affordable prices is a fundamental prerequisite to fulfill healthcare needs of a population [2]. The first Essential Medicines List (EML) by the WHO was introduced in 1977 [3]. However, Sri Lanka implemented a National Hospital Formulary (NHF) to rationalize medicines use and contain cost in the 1950s [4]. Thus, Sri Lanka became one of the first countries in the world to demonstrate that limiting the number of medicines procured and used based on healthcare needs, efficacy and safety could reduce health expenditure and increase the availability of medicines without compromising on quality of healthcare [5]. Since then, Sri Lanka has adopted a Sri Lanka EML in addition to its NHF [6]. An extensive network of government-sponsored hospitals and a fee-levying private healthcare system operates in parallel and the latter provides healthcare services to nearly 40 to 50% of the population in Sri Lanka [7, 8]. Therefore, availability of EMs in community fee-levying pharmacies at affordable prices has a significant impact on equitable delivery of healthcare services in Sri Lanka particularly with increased burden of out-of-pocket expenditure on healthcare on already disadvantaged populations [9, 10].

Publications on availability and affordability of medicines in Sri Lanka prior to implementation of pricing regulations from 2016 is limited to a few studies conducted in limited demographic areas or particular groups of medicines [11–13]. The reported overall availability of medicines in these studies vary from 70 to 75% and originator brands (OBs) are more expensive than the generics. The present study examined the availability, prices, and affordability of 100 EMs in community pharmacies across the country, comparing with number of registered products in 2015 and 2021. The results provide valuable insight into the situation that prevailed before implementation of the price regulations in 2016 and 2017 with a capped pricing system subsequent to the new National Medicines Regulatory Authority (NMRA) Act enforced in 2015 [14, 15]. Comparison of the number of products registered in 2015 with that of 2021 provide an indication on the effect of NMRA Act of 2015 and subsequent regulations on the number of products registered.

## Materials and methods

### Study design and setting

A descriptive cross-sectional national survey including all 25 administrative districts of the 9 provinces in the country was conducted from July to September 2015 to assess the availability, prices, and affordability of 100 selected EMs.

Community pharmacies of all three types in Sri Lanka; namely, ‘Rajya Osusala’ outlets owned and operated by the State Pharmaceuticals Corporation (SPC), SPC franchise pharmacies and privately owned pharmacies were included. The SPC franchise pharmacies are also privately owned but these are operated in keeping with the standards stipulated by the SPC. Selection of pharmacies from each district was done according to the WHO and Health Action International Manual (WHO/HAI) [16]. A multistage cluster sampling approach was followed. The latest lists of ‘Rajya Osusala’ outlets and SPC franchise pharmacies was obtained from the SPC and the list of registered private pharmacies were obtained from the National Medicines Regulatory Authority (NMRA) of Sri Lanka. Administrative districts were chosen as the sampling unit. The main public hospital in each survey area was used to anchor the sample. One pharmacy from each category within three kilometres from main public hospital was chosen.

In districts with a population exceeding 1 million, 2 sets of samples were collected, depending on the availability of the different categories of pharmacies. If Rajya Osusala outlets were not present, an additional privately owned pharmacy was selected. District wise number of pharmacies included in the study is given in Supplementary Table 1.

Ethics approval was obtained from the Ethics Review Committee, Faculty of Medicine, University of Colombo (ERC-15-189) and administrative approvals were obtained from Director General Health Services, Chairman SPC, Chairman NMRA and proprietors of the privately owned pharmacies.

### Selection of survey medicines

Hundred medicines were selected based on commonly prescribed medicines in outpatient settings, healthcare needs in Sri Lanka and inclusion in global/national treatment guidelines. These included 14 medicines from the Global Core List (GCL), 16 from the Regional Core List–Southeast Asia Region (RCL-SEARO) and 70 from the SL-EML of 2013. All the 100 medicines were in the SL-EML of 2013 [17].

### Definitions and outcome measures

Mean availability of all medicines, mean availability of each medicine, the number of brands available, and the availability of the originator brand (OB) was examined. Availability in different categories of pharmacies and provinces were compared. Availability was defined as the percentage of medicines available on the day of the survey. This was categorised as high, fairly high, low and very low if the average availability was over 80%, 50–80%, 30–49% and less than 30% respectively according to the WHO availability index [18].

For price comparison, the WHO/HAI method for comparing local prices with supplier median unit price (USD) listed in Management Sciences for Health Drug Price Indicator Guide (international reference price (IRP)) was used [19]. Indicators used were Median price, Median Price Ratio (MPR) of the median price, MPR of OB, MPR of lowest priced generic (LPG), MPR ratio of the LPG and OB. The MPR was defined as the ratio between the median price of available medicines and the international reference price (IRP). The median price converted to USD based on the average conversion rate given by the Central Bank for 2015 was used to calculate the IRP (1 USD = 144.4606 LKR). MPR below 3 was considered acceptable [16]. If an OB could not be identified, data on availability and price of OB for that medicine could not be collected.

Affordability was assessed using median price and the Defined Daily Dose (DDD) [20]. For medicines without a DDD, standard dose recommended for an adult or a child in 2–5-year age group weighing 10 kg as given in the British National Formulary (BNF 80: September 2020-March 2021) was used. The number of daily wages required by the lowest paid unskilled government worker in 2015 to acquire medicines for a month for chronic conditions or a week/standard course of therapy for acute conditions was calculated. Treatments that cost one days’ income or less were considered affordable. Data on daily wage of a lowest paid government worker was obtained from the labour statistics published by the Department of Labour, Ministry of Labour and Trade Union Relations Sri Lanka in 2015 [21].

### Date collection and analysis

A piloted data collection tool developed using the medicine price data collection form of WHO/ HAI manual was used by doctors trained as data collectors, who worked in pairs to minimise observer biases and errors. Data collection forms were checked for completeness periodically. Data was double entered for verification. Database of registered medicines was obtained from the NMRA in 2022 and the number of products registered in 2015 and 2021 were extracted from it. Data was entered and analysed using both SPSSv.20 and Microsoft Excel according to WHO/HAI guidelines. Comparisons of availability between provinces and different categories of pharmacies were made using one-way ANOVA and Student T-Test, with significance set at p < 0.05 at a 95% confidence interval.

## Results

Eighty pharmacies were selected from the 24 survey areas (Supplementary Table 1). All were surveyed for availability and affordability of the 100 medicines.

### Availability of medicines

The island-wide mean availability was 85.43% (± 12.31). Availability in the Northern province (69.38 ± 21.18%) was significantly lower compared to other 8 provinces which was more than 80% (p = 0.008). Availability in ‘Rajya Osusala’ outlets, SPC franchise pharmacies, and privately owned pharmacies were 87.16 ± 6.22%, 88.5 ± 8.78%, and 82.57 ± 15.73% respectively (Table 1) and were not significantly different between categories (p = 0.053). The average availability of medicines from the global core list (n = 14), regional core list (n = 16) and SL-EML (n = 70) were 85.21 ± 1.65%, 90.31 ± 1.77%, and 85.7 ± 10.08% respectively and were not significantly different between groups (p > 0.05) (Table 1).

**Table 1:** Mean availability of medicines in all pharmacies, SPC outlets, SPC franchise pharmacies and privately owned pharmacies

	All pharmacies n= 80	‘Rajya Osusala’ n = 19	SPC franchise n = 25	Privately owned n = 36	p
Mean availability (%)	85.43 ± 12.31	87.1 ± 6.221	88.5 ± 8.787	82.57 ± 15.738	0.145
Mean aavailability of GCL medicines (%) n = 14	85.21 ± 1.65	83.79 ± 1.195	89.29 ± 0.978	83.21 ± 2.137	0.132
Mean aavailability RCL-SEARO medicines (%) n = 16	90.31 ± 1.771	91.44 ± 1.422	92.44 ± 1.318	88.38 ± 2.136	0.327
Mean aavailability SL-EML medicines (%) n = 70	85.7 ± 10.079	88.41 ± 4.545	88.76 ± 7.225	82.31 ± 12.98	0.150

*GCL – Global core list; RCL-SEARO – Regional Core List South East Asian Region; SL – EML – Sri Lanka Essential Medicine List; SPC – State Pharmaceutical Cooperation*

Only four medicines had availability in less than 50% of the pharmacies (i.e. nystatin oral suspension (100,000 IU/ml) (8.75%), ceftriaxone 1 g vial for injection (13.75%), tramadol hydrochloride 50 mg tablet/capsule (31.25%), carbimazole 5 mg tablet/capsule (38.75%). Seventy-nine of the medicines were available in more than 80% of the pharmacies. Of these 74.6% (n = 59) had an availability of more than 90% (Table 2). Of the 100 medicines surveyed, 37 medicines were available in all SPC outlets, and 29 were available in all SPC franchise pharmacies whilst only 1 medicine was available in all privately owned pharmacies.

**Table 2:** Percentage availability of medicines, availability of the originator brand, number of generic brands in the market and number of registered brands in 2015 and 2021

Medicine	Percentage availability					Number of brands in the market	Number of registered products			
	All (n = 80)	RO (n = 19)	SPC-FP (n = 25)	PO (n = 36)	OB					
							2015	2021	2015 All*	2021 All*
1. Acetylsalicylic acid 75 mg Cap/Tab	88.8	84.2	91.7	89.2	3.8	12	7	6	10	10
2. Aciclovir 200 mg Cap/Tab	88.8	89.5	95.8	83.8	6.3	8	8	6	11	9
3. Alendronate 70 mg Cap/Tab	78.8	84.2	79.2	75.7	7.5	9	6	4	8	4
4. Amiodarone 100 mg Cap/Tab	61.3	47.4	70.8	62.2	6.3	3	1	4	3	8
5. Amitriptyline 25 mg Cap/Tab	91.3	94.7	91.7	89.2	1.3	5	2	1	2	2
6. Amlodipine 5 mg tab	93.8	100	95.8	89.2	1.3	20	16	17	24	24
7. Amoxicillin 125 mg/5ml OS	96.3	100	95.8	94.6	62.5	14	12	4	12	4
8. Amoxicillin 250 mg Cap/Tab	96.3	100	95.8	94.6	45	16	17	8	29	17
9. Amoxicillin + Clavulanic acid 375 mg Cap/Tab	95	89.5	100	94.6	71.3	16	8	14	18	31
10. Atenolol 50 mg Cap/Tab	96.3	94.7	100	94.6	2.5	7	8	9	17	12
11. Atorvastatin 10 mg Cap/Tab	96.3	100	100	91.9	5	24	50	34	63	48
12. Beclomethasone 100mcg/dose MDI	88.8	79	87.5	94.6	0	8	3	5	8	8
13. Benzhexol (Trihexyphenidyl) 2 mg Tab	87.5	94.7	87.5	83.8	2.5	6	2	0	2	0
14. Benzyl benzoate 25% Cream	61.3	63.2	79.2	48.7	56.3	3	0	0	0	0
15. Betahistine 8 mg Cap/Tab	91.3	94.7	100	83.8	5	9	6	8	9	10
16. Bisacodyl 5 mg Suppository	83.8	89.5	83.3	81.1	51.3	4	3	2	3	3
17. Captopril 25 mg Cap/Tab	93.8	84.2	100	94.6	5	6	6	5	6	6
18. Carbimazole 5 mg Cap/Tab	38.8	31.6	54.2	32.4	1.3	4	4	4	5	5
19. Cefalexin 125 mg/ml Suspension	92.5	84.2	100	91.9	10	10	2	2	2	2
20. Ceftriaxone injection 1 g/vial PS	13.8	5.3	20.8	13.5	8.8	3	16	5	20	6
21. Cefuroxime axetil 250 mg Cap/Tab	95	100	100	89.2	11.3	20	18	24	31	38
22. Cetirizine 10 mg Cap/Tab	95	89.5	95.8	97.3	11.3	17	30	28	30	28
23. Ciprofloxacin 0.3% Ear Drops	62.5	36.8	70.8	70.3	12.5	7	10	6	10	6
24. Ciprofloxacin 500 mg Cap/Tab	95	100	91.7	94.6	1.3	13	17	12	29	16
25. Clarithromycin 250 mg Cap/Tab	91.3	94.7	95.8	86.5	18.8	15	15	21	19	24
26. Clonazepam 500 µg Cap/Tab	71.3	73.7	87.5	59.5	33.8	4	0	4	2	7
27. Clotrimazole 1% Cream	91.3	100	95.8	83.8	1.3	12	15	12	15	13
28. Cloxacillin 500 mg Cap/Tab	95	89.5	95.8	97.3	0	7	4	3	8	8
29. Clopidogrel 75 mg Cap/Tab	96.3	94.7	95.8	97.3	8.8	20	46	24	46	24
30. Cotrimoxazole 400 mg + 80 mg Cap/Tab	70	68.4	58.3	78.4	8.8	5	5	3	7	3
31. Co-trimoxazole 40 + 200 mg/5ml OS	61.3	47.4	66.7	64.9	2.5	4	1	1	1	1
32. Condom	80	68.4	87.5	81.1	55	12				
33. Dextrose 50% IVS	82.5	100	66.7	83.8	NI	8	1	3	7	5
34. Diazepam 5 mg Cap/Tab	91.3	100	95.8	83.8	1.3	3	1	1	2	1
35. Diclofenac SR 50 mg Cap/Tab	96.3	94.7	100	94.6	2.5	23	31	28	38	32
36. Diethylcarbamazine citrate 50 mg Cap/Tab	68.8	79	79.2	56.8	3.8	4	0	2	0	2
37. Diltiazem 60 mg Cap/Tab	96.3	94.7	100	94.6	0	12	5	4	11	12
38. Domperidone 10 mg Cap/Tab	98.8	100	100	97.3	61.3	29	25	19	26	19
39. Doxycycline 100 mg Cap/Tab	96.3	94.7	100	94.6	2.5	11	8	8	8	8
40. Enalapril 5 mg Cap/Tab	97.5	100	100	94.6	12.5	9	4	11	7	12
41. Erythromycin 250 mg Cap/Tab	98.8	100	95.8	100	35	16	2	3	7	5
42. Ferrous sulphate 200 mg Cap/Tab	92.5	100	91.7	89.2	NI	10	3	1	3	1
43. Fluoxetine 20 mg Cap/Tab	96.3	100	95.8	94.6	1.3	13	8	7	8	7
44. Folic Acid 1 mg Cap/Tab	97.5	100	100	94.6	1.3	12	3	5	3	5
45. Furosemide 40 mg Cap/Tab	93.8	94.7	100	89.2	81.3	4	0	4	0	5
46. Fusidic acid 1.0% Eye Drop	78.8	89.5	83.3	70.3	40	4	0	2	0	2
47. Gentamicin 0.3% Eye Drop	63.8	52.6	62.5	70.3	6.3	10	11	6	11	6
48. Glibenclamide 5 mg Cap/Tab	92.5	94.7	100	86.5	76.3	8	8	3	8	3
49. Gliclazide 80 mg Cap/Tab	96.3	94.7	95.8	97.3	15	19	24	28	30	36
50. Glyceryl trinitrate 500 micrograms Cap/Tab	65	89.5	58.3	56.8	12.5	8	2	5	2	5
51. Haloperidol 1.5 mg Cap/Tab	88.8	100	87.5	83.8	1.3	5	1	2	2	4
52. Hydrochlorothiazide 50 mg Cap/Tab	91.3	100	87.5	89.2	2.5	5	0	2	0	3
53. Hydrocortisone 1% Cream	95	100	91.7	94.6	1.3	13	7	10	7	10
54. Hydroxychloroquine 200 mg Cap/Tab	77.5	89.5	83.3	67.6	1.3	7	3	6	3	6
55. Ibuprofen 200 mg Cap/Tab	96.3	100	91.7	97.3	60	9	8	5	12	7
56. Insulin (Isophane) 100IU/ml solution for injection	76.3	79	91.7	64.9	22.5	8	2	3	2	3
57. Insulin (Soluble) 100IU/ml solution	56.3	52.6	62.5	54.1	32.5	10	1	2	1	2
58. Isosorbide Mononitrate 60 mg SR Tab	92.5	100	95.8	86.5	0	12	3	3	7	7
59. Lactulose 3.35 g/5ml OS	96.3	94.7	100	94.6	32.5	23	5	10	14	17
60. Levodopa + Carbidopa 275 mg Cap/Tab	90	100	91.7	83.8	50	10	5	2	6	3
61. Levothyroxine 50 mcg Cap/Tab	97.5	100	100	94.6	NI	19	2	8	4	11
62. Lithium carbonate 250 mg ER Tab	77.5	89.5	91.7	62.2	5	9	1	2	2	4
63. Losartan 50 mg Tab	97.5	100	95.8	97.3	3.8	40	32	31	43	37
64. Mebendazole 500 mg Cap/Tab	98.8	100	100	97.3	62.5	12	4	5	9	9
65. Metformin 500 mg Cap/Tab	93.8	100	87.5	94.6	61.3	26	40	38	52	41
66. Methotrexate 2.5 mg Cap/Tab	85	84.2	95.8	78.4	NI	7	3	4	3	4
67. Metoclopramide 10 mg Cap/Tab	90	89.5	95.8	86.5	10	7	5	2	5	2
68. Metronidazole 200 mg Cap/Tab	96.3	100	100	91.9	53.8	8	12	7	15	8
69. Miconazole 2% Cream	92.5	100	91.7	89.2	33.8	21	10	12	10	12
70. Nalidixic acid 300 mg/5ml OS	53.8	36.8	66.7	54.1	NI	3	1	1	1	1
71. Nifedipine ER 20 mg Tab	96.3	100	95.8	94.6	1.3	13	4	7	7	8
72. Nitrofurantoin 50 mg Cap/Tab	85	89.5	79.2	86.5	1.3	6	1	0	1	0
73. Norethisterone 5 mg ER Tab	97.5	100	95.8	97.3	72.5	11	6	4	6	4
74. Normal saline 0.9% Solution	95	100	95.8	91.9	NI	13	7	11	7	11
75. Nystatin 100,000 units/mL OS	8.8	10.5	8.3	8.1	0	3	0	0	0	0
76. OCP - Ethinylestradiol + Levonorgestrel 0.03 mg + 0.15 mg Cap/Tab	76.3	68.4	87.5	73	5	5	5	3	5	3
77. Olanzapine 5 mg Tab	93.8	100	100	86.5	2.5	17	11	7	16	8
78. Omeprazole 20 mg Cap/Tab	97.5	94.7	100	97.3	0	23	35	31	35	32
79. Oral Rehydration Salts 1000ml OS	91.3	100	87.5	89.2	81.3	6	5	4	5	4
80. Paracetamol 120 mg/5ml OS	93.8	89.5	100	91.9	65	7	5	6	6	6
81. Paracetamol 500 mg Cap/Tab	97.5	100	100	94.6	83.8	20	26	18	26	18
82. Phenoxymethylpenicillin 250 mg Cap/Tab	81.3	94.7	75	78.4	2.5	4	1	1	3	1
83. Phenytoin 100 mg Cap/Tab	85	94.7	91.7	75.7	1.3	4	4	2	4	3
84. Prazosin 1 mg Cap/Tab	87.5	94.7	87.5	83.8	3.8	8	4	4	4	5
85. Prednisolone 5 mg Cap/Tab	97.5	100	100	94.6	0	21	8	7	9	11
86. Promethazine 25 mg Cap/Tab	82.5	89.5	91.7	73	0	6	3	2	4	4
87. Propranolol 40 mg Cap/Tab	96.3	100	100	91.9	0	5	1	2	1	2
88. Ranitidine 150 mg Cap/Tab	88.8	68.4	100	91.9	13.8	6	7	5	8	5
89. Risperidone 2 mg Cap/Tab	80	68.4	91.7	78.4	1.3	15	9	7	13	11
90. Salbutamol 2 mg Cap/Tab	96.3	100	95.8	94.6	51.3	12	2	8	4	10
91. Salbutamol 100mcg/dose MDI	93.8	94.7	100	89.2	60	5	5	8	9	12
92. Simvastatin 10 mg Cap/Tab	80	79	87.5	75.7	1.3	9	9	4	11	5
93. Sitagliptin 50 mg Cap/Tab	87.5	89.5	95.8	81.1	2.5	17	4	19	5	26
94. Spironolactone 25 mg Cap/Tab	91.3	94.7	91.7	89.2	57.5	6	5	5	5	5
95. Theophylline 125 mg ER Cap/Tab	90	84.2	95.8	89.2	0	13	2	1	3	3
96. Timolol 0.5% Eye Drop	83.8	79	95.8	78.4	12.5	15	8	3	8	4
97. Tolbutamide 500 mg Cap/Tab	93.8	89.5	100	91.9	NI	5	3	0	3	0
98. Tramadol 50 mg Cap/Tab	31.3	84.2	12.5	16.2	3.8	13	13	2	17	2
99. Valproic-Acid 200 mg Cap/Tab	86.3	89.5	83.3	86.5	33.8	11	5	7	6	9
100. Warfarin 1 mg Cap/Tab	96.3	100	100	91.9	NI	6	2	3	3	5

Cap/tab-Capsule or tablet; ER; Extended release; IVS- Intravenous solution; MDI; Metered Dose Inhaler; NI – Not identified; OB- Originator brand’ OCP- oral contraceptive pill; OS – Oral suspensions; PO – Privately owned pharmacies; PS- powder for solution RO – “Rajya Osu sala”; SI – solution for injection; SPC-FP – State pharmaceutical cooperation franchise pharmacies, SR- Slow released

*The number of registered products of all strength of the specified dosage form

The OB was identified in 92 medicines and their availability was examined. The mean availability was 20.23% (± 25.46) and less than 30% for 69.56% (n = 64) of the medicines. The OB of following 9 medicines (15.09%) were not available in any of the pharmacies; beclomethasone metered dose inhaler (100mcg/dose), cloxacillin capsule 500 mg, diltiazem tablet 60 mg, isosorbide mononitrate slow-release tablet 60 mg, nystatin oral solution 500,000 IU, omeprazole capsule 20 mg, prednisolone tablet 5 mg, promethazine tablet 25 mg, propranolol tablet 40 mg and theophylline extended-release tablet 125 mg.

The median number of generic brands of the selected products in the market was 9 (range 2–44) and 52 medicines had less than 10 generic brands while 13 had 20 or more generic brands in the market. However, the median number of registered products of that specific dosage form and strength in 2015 was 5 and 72.72% (n = 72) of the medicines had more products available in the market than the number registered. Less than half of the registered number of products of atorvastatin 10 mg tablets, ceftriaxone 1 g powder for injection and clopidogrel 75 mg tablets were available in the market. Comparing the number of registered products in 2015 to 2021 showed no significant reduction in the number of registered products, from the year 2015 (median 5, average 8.27) to 2021 (median 5, average 7.59) (p = 0.15) (Table 2).

### Prices of medicines

**Fig. 1:**
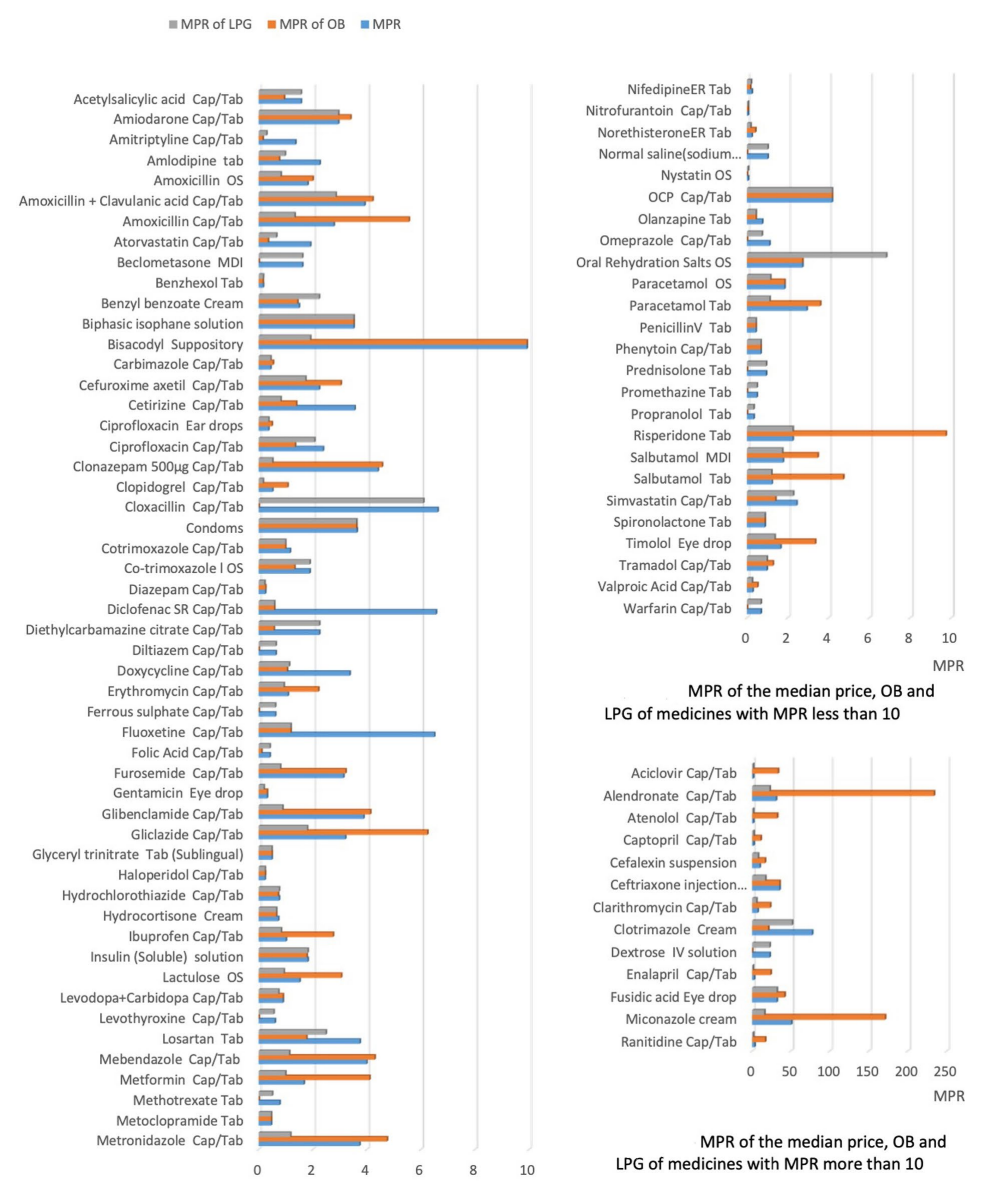
Median price ratio (MPR) of the median price, lowest price generic and originator brand of medicine

The MPR was calculated for 89 medicines (89%) for which the IRP was available. The MPR of 30 (33.7%) medicines was less than 1. The median MPR of LPG was 0.98 and ranged from 0.04 to 51.42. The MPR of 13.48% of LPGs was more than 3. The MPR of the OB ranged from 0.04 to 233.54 and 37.07% of the OBs were more than 3 times the IRP. The OB of 12 (13.48%) medicines had an MPR of over 10. Except for 23 medicines (25.84%), the OB was more expensive than the LPG with a median percentage price difference of 26.77%. Comparison of MPR of the median price, OB and LPG is illustrated in Fig. 1(a) and 1(b). The median price, median price of OB, median price of LPG, MPR of OB, MPR of LPG and the ratio between MPR of OB and LPG for all medicine are given in Supplementary Table 2.

**Table 3:** Duration of therapy, defined daily dose, total cost of treatment and number of daily wages of a lowest paid unskilled government worker required to purchase the course of treatment of unaffordable medicines

Condition	Medicine	Duration	DDD*	Total Cost in USD	Number of Daily wages
Chronic Conditions					
AIIRD	Hydroxychloroquine 200 mg Cap/Tab	30 days	0.516 g	13.39	1.9658
Arrythmia	Amiodarone 100 mg Cap/Tab	30 days	0.2 g	13.50	1.9810
Diabetes mellitus	Insulin (isophane) 100IU/ml solution	30 days	40U	14.62	2.1456
	Insulin (Soluble) 100IU/ml solution	30 days	40U	14.92	2.1895
	Sitagliptin 50 mg Cap/Tab	30 days	0.1 g	16.72	2.4534
Epilepsy	Clonazepam 500 µg Cap/Tab	30 days	8 mg	66.45	9.7528
Hypertension	Losartan 50 mg Cap/Tab	30 days	50 mg	8.10	1.1886
Parkinson Disease	Levodopa + Carbidopa 275 mg Cap/Tab	30 days	0.6 g	13.03	1.9118
Acute Conditions					
Bacterial infection	Ceftriaxone injection 1 g/vial Powder for solution	7 days	2 g	29.93	4.3928

*Standard dose recommended for an adult or a child in 2–5-year age group weighing 10 kg as given in the British National Formulary (BNF) where DDD was not known

AIIRD- autoimmune inflammatory rheumatic diseases; DDD - defined daily dose; g- gram; mg – milligram; ER; Extended release;; LKR – Sri Lankan Rupees ; U – unit; tab- tablet; cap – capsule

#### Affordability

**Fig. 2:**
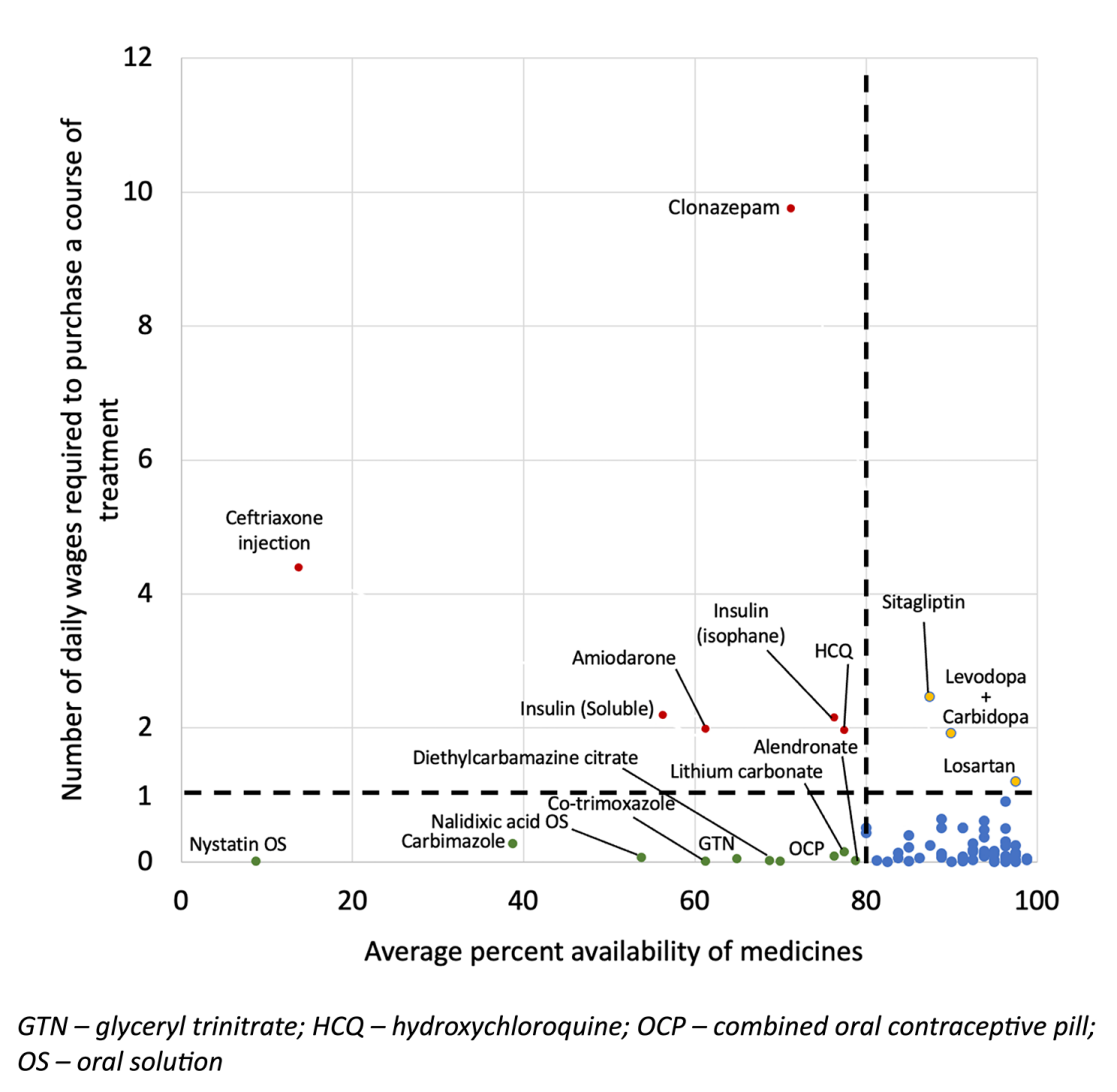
Affordability and average percentage availability of 85 medicines examined for affordability

Out of the 100 medicines, 85 medicines were examined for affordability for which a standard treatment regimen was available. Of these, 89.41% (n = 76) were affordable. Unaffordable medicines which required more than a single daily wage to purchase a standard course of treatment or month’s supply of medicines are listed in Table 3. Six out of these 9 unaffordable medicines had less than 80% average availability. Figure 2 illustrates the affordability and the availability of the 85 medicines examined for affordability. The affordability data of all 85 medicines are given in Supplementary Table 3.

## Discussion

This is the first Island-wide study conducted in Sri Lanka, providing data on 100 EMs showing availability and affordability of medicines used for a wide range of indications, including both communicable and non- communicable diseases. The findings fare well, compared to many other countries with similar economies (Table 4). Similar to previously published studies in Sri Lanka, the overall availability of the EMs was high and the availability of OBs was very low as per the WHO availability index [11, 12]. Studies show that other low and low-middle income countries (LMICs) such as Afghanistan, Armenia, Georgia, Malavi, Nepal, India, and Pakistan have poor availability of EMs of less than 50% [22–24] (Table 4). In certain countries like Mongolia (76.7%) [25] and Brazil (70%) [24] availability of EM were better in private pharmacies compared to the public sector (Table 4). However, we found no such variation between state-owned and privately owned pharmacies in Sri Lanka in the present study. Lower availability OBs probably reflects lower market for OBs due the higher cost and the national policies that promote generic medicines and generic prescribing [26]. Furthermore, notably lower availability of medicines in the Northern province (69.38%) compared to the rest of the country warrants further evaluation and remedial measures.

**Table 4:** Summary of availability, prices and affordability of medicines in Sri Lanka and other low and low-middle income countries from previously published studies

Reference	Country(s) and year of study	Medicines surveyed	Study setting	Mean availability (%)		Mean MPR		Unaffordable medicines
				**Overall**	**OB**	**LPG**	**OB**	
Senarathna, S.M., et al., 2011. [12]	Sri Lanka 2003, 2006 & 2009	28 essential medicines	Community pharmacies	75	50	0.44	4.72	Metformin 500 mg tablet** Carbamazepine 200 mg tablet**
Balasubramaniam, R., et al., 2014 [11]	Sri Lanka 2009	25 essential medicines for children	Privately owned pharmacies			1.35	3.7	3 out of 5 Salbutamol and beclomethasone, carbamazepine syrup
Dabare PRL et al., 2014 [13]	Sri Lanka 2013	50 essential medicines for NCDs	Public and private healthcare facilities, community pharmacies	70.1	25.9			33% of medicines assessed for affordability^#^ (n = 14)
Mendis S et al. 2007 [22]	Bangladesh	32 medicines used to treat chronic diseases	Public and private medicine outlets (hospitals or dispensaries)	30.0**	10.0	1.14	1.31	4 out 5 - Aspirin + atenolol + ACE inhibitor + statin - glibenclamide/ metformin, beclomethasone + salbutamol inhaler
	Brazil			70.0	65.0	6.34	16.15	
	Malawi			37.5	0.0	4.51	6.28	
	Nepal			28.4	0.0	2.05	3.53	
	Pakistan			31.7	51.7	1.64	1.72	
	Sri Lanka.			78.6	4.1	1.05**	2.23	
Cameron, A., et al., 2009 [1]	36 countries	15 medicines	Not specified. It is a secondary analysis of data from 45 WHO/HAI surveys	75·1^$ P^ 38·3^$ Pu^	22·3- LIC 43·7 - LMIC	9·61^$ P^ 6·84^$ Pu^	21·28^$^	1 out of 4 Ranitidine 150 mg^$^
Kotwani A., 2011 [35]	India 2011	50 medicines	Public and private sector facilities	41.3^P^ 23.2 ^Pu^		2.83 ^P^	4.41^P^	
Dorj, G., et al., 2018 [23]	Mongolia 2016	30 medicines + 1 device	Public and private sector pharmacies	76.7		2.45^P^ 1.95^Pu^		Amoxicillin Clavulanic Acid Suspension 125–31.25 mg/5 ml
Saeed A., et al., 2019 [20]	Pakistan 2016–2017	50 (46 were in EM)	Public sector and private sector pharmacies	20.3%	55%*	0.42 - 19.96	0.58–60.63	3 out of 12 Isophane insulin, regular insulin, omeprazole**
Babar Z-U-D, et al., 2019 [21] Year of study – 2018, in 17 countries	Afghanistan	4 essential diabetes medicines	Primary care pharmacies	42	50			2 out of 4 in Low-income countries Metformin and insulin (OB)
	Armenia			33	25			
	Bangladesh			38	0			
	Egypt			46	17			
	Georgia			29	33			
	India			44	25			
	Nepal			38	0			
	Pakistan			88	75			
	Sri Lanka			63	67			
	Tanzania			38	25			
Kasonde L., et al. 2019 [36]	Bangladesh 2019	61 medicines	Public hospitals, private retail pharmacies, and private clinics in 6 regions	63*	4*	1.70	3.69	4 out of 16 Metformin; bisoprolol; atorvastatin; diclofenac (OB)

*in retail pharmacies

**availability of the lowest priced generic

^$^ in the Southeast Asian region

^#^Amiodarone. Beclometasone 200 µg capsules, Carvedilol, Glyceryl trinitrate 500 µg tablet, Insulin (soluble), Intermediate acting insulin, Ipratropium bromide, Levodopa- carbidopa 100 + 25 mg tablet, Methyldopa, Phenytoin, Salbutamol (MDI), Simvastatin 20 mg, Sodium valproate 200 mg tablet, valproate 200 mg/5 ml syrup

^P^ in private pharmacies

^Pu^ in public sector pharmacies

*LIC– low-income countries; LMIC – low middle-income countries; LPG – lowest price generic; MPR – Median price ratio; OB - Originator brand*

Some findings of the present study are not congruent with previous reports possibly due to differences in sampling of pharmacies, medicines selected for the different surveys and year of surveys. For instance, a survey of 25 key EMs for children in privately owned pharmacies in Sri Lanka done in 2009 report a MPR of more than 1 for the LPG for 75% of the medicines [11]. However, similar to the findings of the present study, another national survey of 50 EMs for non-communicable diseases (NCDs) in public and private pharmacies done in 2013 and a study in one geographical area of 28 medicine done from 2003 to 2009, showed that most generic medicines had an MPR of less than 1. One third of the MPRs in the present study are less than 1, indicating that these medicines are cheaper than in the reference countries and that pricing in community pharmacies including privately owned pharmacies is appropriate. The MPR of 37% of the OBs exceeded 3 while 13.4% had an MPR of over 10, which could be due to less stringent regulation of markups in Sri Lanka. Furthermore, overall the OB were 26.77% more expensive than the LPGs. Interestingly some OBs were less expensive than the LPG. These observations could be due to variety of reasons including reduction of the price after patent expiry and competitive pricing because of the higher number of generic equivalents in the market [27] .

A previous national survey of medicines for NCDs reported only 67% of the surveyed medicines to be affordable whereas 89.41% of the medicines in the present study were affordable [13]. Amiodarone and insulin were found to be unaffordable in both studies. However, Sri Lanka fared better in 2015 compared to most other LMIC where medicines are largely unaffordable [1, 28]. Although many factors affect the prices of medicines, the presence of state-owned Rajya Osusala outlets in most of the provinces offering medicines at a competitive affordable price may have an implicit price regulatory effect contributing to comparatively lower prices of medicines noted in all 3 categories of pharmacies.

This study revealed that there were more products available in the market than the number registered in 2015. This could be due to a multitude of reasons including delays in processing of applications for re-registrations, entry of unregistered products into the market due to lack of monitoring and stringent control of un-authorized sale of pharmaceuticals. This raises many concerns, as unregistered medicines have the potential to compromise quality and safety of medicines due to their unregulated nature [29–31]. Furthermore, the present study identified some medicines had a large number of products registered with less than 50% of them being available in the market. Such major disparities makes monitoring and regulation even more challenging particularly in a resource limited setting such as Sri Lanka. The NMRA Act requires an applicant to demonstrate a justifiable need for registration of a new product when other generic equivalents already exist in the market [32]. This “need clause” in the NMRA Act was expected to limit the number of brands in the market. However, the overall average number registered products has not changed significantly from 2015 to 2021. This is probably due to the previously registered products remaining in the registry with restrictions imposed only on entry of new products. Nevertheless, evidence shows that competition could lower prices of medicines within a class, and it may anchor the prices of new medicines below what they would have been priced in the absence of such competition [33]. Furthermore, the effect of brand-brand competition on pricing is also determined by the relative quality of medicines [34]. Therefore, this multiprong strategy of controlling entry of new products and price regulation could potentially improve the quality and safety of medicines and control the market entry price.

This study presenting data on 100 EMs from community pharmacies provide nationally representative data for standardized international comparison and gives a useful baseline to assess the effects of pricing and registration regulations enforced in Sri Lanka after the establishment of the NMRA in 2015. A follow-up survey of these 100 medicines is needed to assess the full extent of the effect these regulations enforced in 2015 and to assess the impact of the current economic crisis in the country. There are a few limitations of the present study. It does not reflect variations in the availability of medicines throughout the year as it is a single point cross-sectional study. Furthermore, it is limited to community pharmacies and thus, does not reflect the overall availability in all healthcare facilities in the country. Although the daily wage of a lowest paid unskilled government worker was used to assess affordability for most of the population, there may be a significant number of daily wage earners and unemployed individuals with a lower income. Hence, this data may not be a true representation of affordability of medicines for the entire population.

## Conclusion

The overall availability of EMs in Sri Lanka for a wide range of indications is high in all categories of community pharmacies. Medicines in Sri Lanka are largely affordable and reasonably priced, although OBs are generally more expensive. There is a marked variability seen in the MPR of medicines in Sri Lanka. There were a higher number of products of each medicine in the market than the number registered in the NMRA in 2015. This is possibly due to the unregulated nature of the pharmaceutical market which should be addressed to ensure quality and safety of medicines in Sri Lanka. However, the median number of registered products has not changed significantly from 2015 to 2021. A follow-up survey to assess the extent of the effect of revised pricing and registration related regulations implemented and to assess the impact of the current economic crisis where availability of several EMs are noted to be unavailable will provide valuable data for implementation of further actions.

### Electronic supplementary material

Below is the link to the electronic supplementary material.

Supplementary Material 1

Supplementary Material 2

Supplementary Material 3

## Data Availability

The datasets used and/or analysed during the current study are available from the corresponding author on reasonable request.

## List of Abbreviations

BNF: British National Formulary
DDD: Defined daily dose
EM: Essential medicines
EML: Essential medicines Llist
GCL: Global core list
HAI: Health Action International
IRP: International reference price
LMIC: Low-middle income countries
LPG: Lowest priced generic
MPR: Median price ratio
NCDs: Non-communicable diseases
NHF: National hospital formulary
NMRA: National Medicines Regulatory Authority
OB: Originator brands
RCL-SEARO: Regional core list–Southeast Asia region
SL-EML: Sri Lanka essential medicines list
SPC: State Pharmaceuticals Corporation
WHO: World Health Organisation
