# Supplementary material for: A national survey on registered products, availability, prices, and affordability of 100 essential medicines in community pharmacies across Sri Lanka
Source: BMC Health Serv Res. 2023 Oct 19;23:1121. doi: 10.1186/s12913-023-10137-y (PMC10585786; doi:10.1186/s12913-023-10137-y)
Supplement: Supplementary file 1 — Supplementary Material 1 [file 12913_2023_10137_MOESM1_ESM.docx]

**Supplementary Table 1:** District wise number of pharmacies selected for the survey

| District | Size of population | Pharmacy Outlets | | | Total |
| --- | --- | --- | --- | --- | --- |
|  |  | Privately  owned | State owned  (‘Rajya Osusala’ – SPC) | SPC Franchise |  |
| 1. Colombo | 2,324,349 | 2 | 2 | 2 | 6 |
| 1. Gampaha | 2,304,833 | 2 | 2 | 2 | 6 |
| 1. Kurunegala | 1,618,465 | 2 | 1 | 2 | 5 |
| 1. Kandy | 1,375,382 | 2 | 1 | 2 | 5 |
| 1. Kalutara | 1,221,948 | 2 | 2 | 2 | 6 |
| 1. Ratnapura | 1,088,007 | 2 | 2 | 2 | 6 |
| 1. Galle | 1,063,334 | 2 | 2 | 2 | 6 |
| 1. Anuradhapura | 860,575 | 1 | 1 | 1 | 3 |
| 1. Kegalle | 840,648 | 1 | NA | 1 | 2 |
| 1. Badulla | 815,405 | 1 | 1 | 1 | 3 |
| 1. Matara | 814,048 | 1 | 1 | 1 | 3 |
| 1. Puttalam | 762,396 | 1 | NA | 1 | 2 |
| 1. Nuwara Eliya | 711,644 | 2 | NA | N/A | 2 |
| 1. Ampara | 649,402 | 1 | 1 | NA | 2 |
| 1. Hambantota | 599,903 | 1 | 1 | 1 | 3 |
| 1. Jaffna | 583,882 | 1 | 1 | 1 | 3 |
| 1. Batticaloa | 526,567 | 2 | NA | N/A | 2 |
| 1. Matale | 484,531 | 1 | NA | 1 | 2 |
| 1. Monaragala | 451,058 | 1 | NA | 1 | 2 |
| 1. Polonnaruwa | 406,088 | 1 | 1 | 1 | 3 |
| 1. Trincomalee | 379,541 | 1 | NA | 1 | 2 |
| 1. Vavuniya | 172,115 | 2 | NA | NA | 2 |
| 1. Kilinochchi | 113,510 | 2 | NA | NA | 2 |
| 1. Mannar | 99,570 | 2 | NA | NA | 2 |
| 1. Mullaitivu | 92,238 |  |  |  |  |
| Total | 20,359,439 | 36 | 19 | 25 | 80 |

NA – Not available; SPC – State Pharmaceuticals Corporation
