# Supplementary material for: A national survey on registered products, availability, prices, and affordability of 100 essential medicines in community pharmacies across Sri Lanka
Source: BMC Health Serv Res. 2023 Oct 19;23:1121. doi: 10.1186/s12913-023-10137-y (PMC10585786; doi:10.1186/s12913-023-10137-y)
Supplement: Supplementary file 2 — Supplementary Material 2 [file 12913_2023_10137_MOESM2_ESM.docx]

**Supplementary table 2: Pricing of Medicines**

| **Medicine- Strength -Dosage form** | **Median price**  **in USD** | **MPR (Median price/IRP)** | **Median price of OB in USD** | **Median price of LPG in USD** | **MPR of OB** | **MPR of LPG** | **Percentage difference between OB and LPG** |
| --- | --- | --- | --- | --- | --- | --- | --- |
| **Medicines from the Global Core List** | | | | | | | |
| 1. Amitriptyline 25 mg Cap/Tab | 0.0380 | 1.3573 | 0.0042 | 0.0080 | 0.1487 | 0.2851 | -47.50 |
| 1. Amoxicillin 250mg Cap/Tab | 0.0486 | 2.7766 | 0.0972 | 0.0233 | 5.5532 | 1.3288 | 317.17 |
| 1. Atenolol 50mg Cap/Tab | 0.0080 | 1.3530 | 0.1905 | 0.0069 | 32.2900 | 1.1765 | 2660.87 |
| 1. Captopril 25mg Cap/Tab | 0.0137 | 1.7993 | 0.0825 | 0.0137 | 10.8507 | 1.7993 | 502.19 |
| 1. Ceftriaxone injection 1 g/vial -powder for solution | 15.0076 | 35.3038 | 15.0111 | 7.1238 | 35.3119 | 16.7579 | 110.72 |
| 1. Ciprofloxacin 500mg Cap/Tab | 0.0640 | 2.3792 | 0.0361 | 0.0554 | 1.3419 | 2.0605 | -34.84 |
| 1. Co-trimoxazole 40+200mg/5ml OS | 0.0079 | 1.8841 | 0.0056 | 0.0079 | 1.3222 | 1.8841 | -29.11 |
| 1. Diazepam 5 mg Cap/Tab | 0.0027 | 0.2396 | 0.0028 | 0.0024 | 0.2457 | 0.2150 | 16.67 |
| 1. Diclofenac SR 50 mg Cap/Tab | 0.0833 | 6.5589 | 0.0073 | 0.0073 | 0.5739 | 0.5739 | 0.00 |
| 1. Glibenclamide 5 mg Cap/Tab | 0.0205 | 3.8768 | 0.0219 | 0.0047 | 4.1256 | 0.8775 | 365.96 |
| 1. Omeprazole 20mg Cap/Tab | 0.0289 | 1.0964 | * | 0.0192 | - | 0.7270 | - |
| 1. Paracetamol 120mg/5ml OS | 0.0118 | 1.8438 | 0.0118 | 0.0073 | 1.8438 | 1.1388 | 61.64 |
| 1. Salbutamol 100mcg/dose MDI | 0.0103 | 1.7713 | 0.0201 | 0.0101 | 3.4708 | 1.7354 | 99.01 |
| 1. Simvastatin 10mg Cap/Tab | 0.0326 | 2.4347 | 0.0187 | 0.0303 | 1.3987 | 2.2690 | -38.28 |
| **Medicines selected from the South East Asia Regional Core List (SEARO)** | | | | | | | |
| 1. Amlodipine 5 mg tab | 0.0354 | 2.2549 | 0.0118 | 0.0153 | 0.7516 | 0.9727 | -22.88 |
| 1. Amoxicillin 125mg/5ml OS | 0.0170 | 1.8092 | 0.0187 | 0.0076 | 1.9939 | 0.8123 | 146.05 |
| 1. Atorvastatin 10 mg Cap/Tab | 0.1019 | 1.9083 | 0.0184 | 0.0347 | 0.3445 | 0.6500 | -46.97 |
| 1. Beclometasone 100mcg/dose MDI | 0.0180 | 1.6114 | * | 0.0180 | - | 1.6114 | - |
| 1. Clotrimazole 15g Cream | 1.0412 | 77.1284 | 0.2777 | 0.6942 | 20.5676 | 51.4189 | -60.00 |
| 1. Diethylcarbamazine citrate 50mg Cap/Tab | 0.0139 | 2.2392 | 0.0035 | 0.0139 | 0.5598 | 2.2392 | -74.82 |
| 1. Doxycycline 100mg Cap/Tab | 0.0646 | 3.3623 | 0.0201 | 0.0216 | 1.0485 | 1.1244 | -6.94 |
| 1. Enalapril 5mg Cap/Tab | 0.0146 | 2.3512 | 0.1463 | 0.0049 | 23.6013 | 0.7837 | 2885.71 |
| 1. Fluoxetine 20mg Cap/Tab | 0.0669 | 6.4934 | 0.0121 | 0.0121 | 1.1794 | 1.1794 | 0.00 |
| 1. Gentamicin 0.3% Eye drop | 0.0680 | 0.3064 | 0.0680 | 0.0416 | 0.3064 | 0.1876 | 63.46 |
| 1. Gliclazide 80 mg Cap/Tab | 0.0711 | 3.2019 | 0.1385 | 0.0397 | 6.2380 | 1.7901 | 248.87 |
| 1. Ibuprofen 200mg Cap/Tab | 0.0069 | 1.0060 | 0.0190 | 0.0057 | 2.7464 | 0.8249 | 233.33 |
| 1. Metformin 500 mg Cap/Tab | 0.0271 | 1.6711 | 0.0664 | 0.0160 | 4.0964 | 0.9855 | 315.00 |
| 1. Metronidazole 200mg Cap/Tab | 0.0250 | 3.7298 | 0.0318 | 0.0078 | 4.7451 | 1.1707 | 307.69 |
| 1. Phenytoin 100mg Cap/Tab | 0.0298 | 0.6648 | 0.0298 | 0.0298 | 0.6648 | 0.6648 | 0.00 |
| 1. Ranitidine 150mg Cap/Tab | 0.0569 | 3.0117 | 0.3141 | 0.0224 | 16.6193 | 1.1826 | 1302.23 |
| **Medicines Selected from the Sri Lanka Essential Medicines List** | | | | | | | |
| 1. Acetylsalicylic acid 75mg Cap/Tab | 0.0231 | 1.5618 | 0.0139 | 0.0231 | 0.9380 | 1.5618 | -39.83 |
| 1. Aciclovir 200mg Cap/Tab | 0.0399 | 1.2319 | 1.0829 | 0.0399 | 33.4223 | 1.2319 | 2614.04 |
| 1. Alendronate 70mg Cap/Tab | 0.7289 | 30.8840 | 5.5116 | 0.5276 | 233.5421 | 22.3542 | 944.66 |
| 1. Amiodarone 100mg Cap/Tab | 0.2256 | 2.9413 | 0.2603 | 0.2256 | 3.3938 | 2.9413 | 15.38 |
| 1. Amoxicillin + Clavulanic acid 250mg Cap/Tab | 0.4581 | 3.9157 | 0.4929 | 0.3332 | 4.2124 | 2.8478 | 47.93 |
| 1. Benzhexol (Trihexyphenidyl) 2mg Tab | 0.0037 | 0.1600 | 0.0035 | 0.0037 | 0.1509 | 0.1600 | -5.41 |
| 1. Benzyl benzoate 25% Cream | 0.0042 | 1.4875 | 0.0040 | 0.0062 | 1.4317 | 2.2312 | -35.48 |
| 1. Betahistine 8mg Cap/Tab | 0.0672 | ** | 0.1770 | 0.0625 | - | - | 183.20 |
| 1. Biphasic isophane 100IU/ml solution for injection | 1.2217 | 3.5107 | 1.2217 | 1.2217 | 3.5107 | 3.5107 | 0.00 |
| 1. Bisacodyl 5mg Suppository | 0.1805 | 9.9165 | 0.1805 | 0.0347 | 9.9165 | 1.9070 | 420.17 |
| 1. Carbimazole 5mg Cap/Tab | 0.0201 | 0.4376 | 0.0243 | 0.0201 | 0.5282 | 0.4376 | 20.90 |
| 1. Cefalexin 125mg/ml suspension | 0.0610 | 9.5338 | 0.1041 | 0.0469 | 16.2693 | 7.3212 | 121.96 |
| 1. Cefuroxime axetil 250mg Cap/Tab | 0.3679 | 2.2338 | 0.4998 | 0.2846 | 3.0346 | 1.7280 | 75.61 |
| 1. Cetirizine 10mg Cap/Tab | 0.0319 | 3.5479 | 0.0125 | 0.0073 | 1.3883 | 0.8098 | 71.23 |
| 1. Ciprofloxacin 0.3% Ear drops | 0.1041 | 0.3602 | 0.1402 | 0.1041 | 0.4850 | 0.3602 | 34.68 |
| 1. Clarithromycin 250mg Cap/Tab | 0.2603 | 6.9416 | 0.8608 | 0.2013 | 22.9534 | 5.3681 | 327.62 |
| 1. Clonazepam 500µg Cap/Tab | 0.1388 | 4.4073 | 0.1437 | 0.0160 | 4.5616 | 0.5068 | 798.13 |
| 1. Clopidogrel 75mg Cap/Tab | 0.1122 | 0.5074 | 0.2360 | 0.0347 | 1.0674 | 0.1570 | 580.12 |
| 1. Cloxacillin 500mg Cap/Tab | 0.0377 | 6.6188 | * | 0.0347 | - | 6.0891 | - |
| 1. Condoms | 0.1157 | 3.6275 | 0.1152 | 0.1152 | 3.6122 | 3.6122 | 0.00 |
| 1. Cotrimoxazole 400mg+80mg Cap/Tab | 0.0139 | 1.1569 | 0.0118 | 0.0118 | 0.9834 | 0.9834 | 0.00 |
| 1. Dextrose 50% IV solution | 0.2430 | 22.2894 | * | 0.2430 | - | 22.2894 | - |
| 1. Diltiazem 60mg Cap/Tab | 0.0237 | 0.6297 | * | 0.0237 | - | 0.6297 | - |
| 1. Domperidone 10mg Cap/Tab | 0.0382 | ** | 0.1192 | 0.0128 | - | - | 831.25 |
| 1. Erythromycin 250mg Cap/Tab | 0.0486 | 1.0798 | 0.0993 | 0.0416 | 2.2059 | 0.9255 | 138.70 |
| 1. Ferrous sulphate 200mg Cap/Tab | 0.0060 | 0.6100 | * | 0.0060 | - | 0.6100 | - |
| 1. Folic Acid 1mg Cap/Tab | 0.0127 | 0.4085 | 0.0031 | 0.0127 | 0.1004 | 0.4085 | -75.59 |
| 1. Furosemide 40mg Cap/Tab | 0.0194 | 3.1349 | 0.0199 | 0.0049 | 3.2133 | 0.7949 | 306.12 |
| 1. Fusidic acid 1.0% Eye drop | 2.6794 | 31.8981 | 3.5124 | 2.6794 | 41.8146 | 31.8981 | 31.09 |
| 1. Glyceryl trinitrate 500 mcg Tab (Sublingual) | 0.0562 | 0.4818 | 0.0562 | 0.0555 | 0.4818 | 0.4759 | 1.26 |
| 1. Haloperidol 1.5mg Cap/Tab | 0.0026 | 0.2274 | 0.0027 | 0.0026 | 0.2334 | 0.2274 | 3.85 |
| 1. Hydrochlorothiazide 50mg Cap/Tab | 0.0037 | 0.7508 | 0.0035 | 0.0037 | 0.7154 | 0.7508 | -5.41 |
| 1. Hydrocortisone 1% Cream | 0.0360 | 0.7219 | 0.0323 | 0.0323 | 0.6478 | 0.6478 | 0.00 |
| 1. Hydroxychloroquine 200mg Cap/Tab | 0.1735 | ** | 0.1331 | 0.1701 | - | - | -21.75 |
| 1. Insulin (Soluble) 100IU/ml solution | 1.2471 | 1.8127 | 1.2217 | 1.2471 | 1.7757 | 1.8127 | -2.04 |
| 1. Isosorbide Mononitrate 60mg slow release Tab | 0.0923 | ** | * | 0.0921 | - | - | - |
| 1. Lactulose 3.35g/5ml OS | 0.0165 | 1.5125 | 0.0333 | 0.0101 | 3.0511 | 0.9285 | 229.70 |
| 1. Levodopa+Carbidopa 275mg Cap/Tab | 0.1996 | 0.8925 | 0.1996 | 0.1630 | 0.8925 | 0.7289 | 22.45 |
| 1. Levothyroxine 50 mcg Cap/Tab | 0.0451 | 0.5976 | * | 0.0416 | - | 0.5516 | - |
| 1. Lithium carbonate 250mg Tab (Prolong Release) | 0.0382 | ** | 0.0311 | 0.0382 | - | - | -18.59 |
| 1. Losartan 50mg Tab | 0.0677 | 3.7392 | 0.0319 | 0.0449 | 1.7642 | 2.4813 | -28.95 |
| 1. Mebendazole 500 mg Cap/Tab | 0.8330 | 3.9875 | 0.8955 | 0.2359 | 4.2865 | 1.1293 | 279.61 |
| 1. Methotrexate 2.5mg Tab | 0.0486 | 0.7725 | * | 0.0312 | - | 0.4966 | - |
| 1. Metoclopramide 10mg Tab | 0.0069 | 0.4597 | 0.0069 | 0.0069 | 0.4597 | 0.4597 | 0.00 |
| 1. Miconazole 2% cream | 0.8781 | 50.4659 | 2.9710 | 0.2707 | 170.7462 | 15.5587 | 997.52 |
| 1. Nalidixic acid 300mg/5ml OS | 0.0320 | ** | * | 0.0313 | - | - | - |
| 1. NifedipineER 20mg Tab | 0.0108 | 0.2334 | 0.0073 | 0.0090 | 0.1581 | 0.1957 | -18.89 |
| 1. Nitrofurantoin 50mg Cap/Tab | 0.0061 | 0.0439 | 0.0061 | 0.0061 | 0.0439 | 0.0439 | 0.00 |
| 1. Norethisterone 5mg Prolong release Tab | 0.0540 | 0.2315 | 0.0944 | 0.0399 | 0.4047 | 0.1711 | 136.59 |
| 1. Normal saline(sodium chloride) 0.9% | 0.0011 | 1.0097 | * | 0.0011 | - | 1.0097 | - |
| 1. Nystatin 100,000 units/mL OS | 0.0021 | 0.0511 | * | 0.0021 | * | 0.0511 | - |
| 1. OCP - Combined Oral Contraceptive Pills (Ethinylestradiol +Levonorgestrel) 0.03mg+0.15mg Cap/Tab | 0.6942 | 4.1691 | 0.6942 | 0.6942 | 4.1691 | 4.1691 | 0.00 |
| 1. Olanzapine 5mg Tab | 0.0694 | 0.7408 | 0.0399 | 0.0403 | 0.4260 | 0.4297 | -0.99 |
| 1. Oral Rehydration Salts 1000ml OS | 0.1527 | 2.7222 | 0.1527 | 0.3825 | 2.7222 | 6.8178 | -60.08 |
| 1. Paracetamol 500mg Tab | 0.0170 | 2.9322 | 0.0208 | 0.0064 | 3.5905 | 1.1011 | 225.00 |
| 1. Penicillin V (Phenoxymethylpenicillin) 250mg Tab | 0.0165 | 0.4258 | 0.0165 | 0.0165 | 0.4258 | 0.4258 | 0.00 |
| 1. Prazosin 1mg Tab | 0.0111 | ** | 0.0080 | 0.0111 | - | - | -27.93 |
| 1. Prednisolone 5mg Tab | 0.0101 | 0.9320 | * | 0.0101 | - | 0.9320 | - |
| 1. Promethazine 25mg Tab | 0.0037 | 0.4806 | * | 0.0037 | - | 0.4806 | - |
| 1. Propranolol 40mg Tab | 0.0035 | 0.3246 | * | 0.0035 | - | 0.3214 | - |
| 1. Risperidone 2mg Tab | 0.0458 | 2.2458 | 0.1985 | 0.0458 | 9.7318 | 2.2458 | 333.41 |
| 1. Salbutamol 2mg Tab | 0.0031 | 1.2217 | 0.0118 | 0.0030 | 4.7203 | 1.1939 | 293.33 |
| 1. Sitagliptin 50mg Tab | 0.2794 | ** | 0.2495 | 0.2707 | - | - | -7.83 |
| 1. Spironolactone 25mg Tab | 0.0383 | 0.8669 | 0.0383 | 0.0383 | 0.8669 | 0.8669 | 0.00 |
| 1. Theophylline ER 125mg Cap/Tab | 0.0191 | ** | * | 0.0191 | - | - | - |
| 1. Timolol 0.5% Eye drop | 0.2943 | 1.6498 | 0.5970 | 0.2430 | 3.3463 | 1.3619 | 145.68 |
| 1. Tolbutamide 500mg Tab | 0.0108 | ** | * | 0.0108 | - | - | - |
| 1. Tramadol 50mg Cap/Tab | 0.0264 | 0.9662 | 0.0347 | 0.0264 | 1.2713 | 0.9662 | 31.44 |
| 1. Valproic Acid 200mg Cap/Tab | 0.0486 | 0.2769 | 0.0896 | 0.0427 | 0.5106 | 0.2433 | 109.84 |
| 1. Warfarin 1 mg Cap/Tab | 0.0202 | 0.6733 | * | 0.0202 | - | 0.6733 | - |

**OB is not available or not specified; ** IRP is not available*

Cap/tab-Capsule or tablet; ER; Extended release; IRP – International reference price; IVS- Intravenous solution; LPG – lowest priced generic; MDI; Metered Dose Inhaler; MPR – median reference price; NI – Not identified; OB- Originator brand; OCP- oral contraceptive pill; OS – Oral suspensions; PO – Privately owned pharmacies; PS- powder for solution; RO – “Rajya Osu sala”; SI – solution for injection; SPC-FP – State pharmaceutical cooperation franchise pharmacies; SR- Slow released
