# Supplementary material for: A national survey on registered products, availability, prices, and affordability of 100 essential medicines in community pharmacies across Sri Lanka
Source: BMC Health Serv Res. 2023 Oct 19;23:1121. doi: 10.1186/s12913-023-10137-y (PMC10585786; doi:10.1186/s12913-023-10137-y)
Supplement: Supplementary file 3 — Supplementary Material 3 [file 12913_2023_10137_MOESM3_ESM.docx]

**Supplementary table 3: Duration of therapy, defined daily dose, total cost of treatment and number of daily wages of a lowest paid unskilled government worker required to purchase the course of treatment**

| **Condition** | **Medicine** | **Duration** | **DDD*** | **Total Cost in LKR** | **Number of Daily wages** |
| --- | --- | --- | --- | --- | --- |
| **Chronic Conditions** |  |  |  |  |  |
| AIIRD | Hydroxychloroquine 200mg Cap/Tab | 30 days | 0.516 g | 1935 | 1.9658 |
|  | Methotrexate 2.5mg Cap/Tab | 30 days | 2.5 mg | 210 | 0.2133 |
|  | Prednisolone 5mg Cap/Tab | 30 days | 10mg | 87 | 0.0884 |
| Anxiety | Diazepam 5 mg Cap/Tab | 30days | 10mg | 23.4 | 0.0238 |
| Arrythmia | Amiodarone 100mg Cap/Tab | 30 days | 0.2g | 1950 | 1.9810 |
| Asthma | Beclomethasone 100mcg/dose MDI | 30 days | 0.8mg | 624 | 0.6339 |
|  | Salbutamol 100mcg/dose MDI | 30 days | 0.8mg | 355.2 | 0.3609 |
|  | Salbutamol 2mg Tab | 30 days | 12mg | 79.2 | 0.0805 |
| Contraception | OCP - Ethinylestradiol +Levonorgestrel 0.03mg+0.15mg Cap/Tab | 28 days | 1 UD | 78 | 0.0792 |
| Depression | Amitriptyline 25 mg Cap/Tab | 30 days | 75mg | 492.75 | 0.5006 |
|  | Fluoxetine 20mg Cap/Tab | 30 days | 20mg | 289.05 | 0.2937 |
|  | Glibenclamide 5 mg Cap/Tab | 30 days | 10mg | 177.6 | 0.1804 |
|  | Gliclazide 80 mg Cap/Tab | 30 days | 60mg | 230.4 | 0.2341 |
|  | Insulin (isophane) 100IU/ml solution | 30 days | 40U | 2112 | 2.1456 |
|  | Insulin (Soluble) 100IU/ml solution | 30 days | 40U | 2155.2 | 2.1895 |
|  | Metformin 500 mg Cap/Tab | 30 days | 2g | 468 | 0.4755 |
|  | Sitagliptin 50mg Cap/Tab | 30 days | 0.1g | 2415 | 2.4534 |
|  | Tolbutamide 500mg Cap/Tab | 30 days | 1.5g | 140.4 | 0.1426 |
| Oedema | Furosemide 40mg Cap/Tab | 30 days | 40mg | 84 | 0.0853 |
|  | Hydrochlorothiazide 50mg Cap/Tab | 30 days | 25mg | 7.95 | 0.0081 |
|  | Spironolactone 25mg Tab | 30 days | 75mg | 496.8 | 0.5047 |
| Endometriosis | Norethisterone 5mg ER Tab | 30 days | 5mg | 233.4 | 0.2371 |
| Epilepsy | Clonazepam 500µg Cap/Tab | 30 days | 8mg | 9600 | 9.7528 |
|  | Phenytoin 100mg Cap/Tab | 30 days | 0.3g | 387 | 0.3932 |
|  | Valproic-Acid 200mg Cap/Tab | 30 days | 1.5g | 52.5 | 0.0533 |
| Glaucoma | Timolol 0.5% Eye Drop | 30 days | 0.1 ml | 127.2 | 0.1292 |
| hyperlipidemia | Atorvastatin 10 mg Cap/Tab | 30 days | 20mg | 880.8 | 0.8948 |
|  | Simvastatin 10mg Cap/Tab | 30 days | 30 mg | 423 | 0.4297 |
| Hypertension | Amlodipine 5 mg Cap/Tab | 30 days | 5 mg | 153 | 0.1554 |
|  | Atenolol 50mg Cap/Tab | 30 days | 75mg | 51.75 | 0.0526 |
|  | Captopril 25mg Cap/Tab | 30 days | 50mg | 118.2 | 0.1201 |
|  | Carbimazole 5mg Cap/Tab | 30 days | 15mg | 261 | 0.2652 |
|  | Diltiazem 60mg Cap/Tab | 30 days | 0.24g | 13.68 | 0.0139 |
|  | Enalapril 5mg Cap/Tab | 30 days | 10mg | 126 | 0.1280 |
|  | Losartan 50mg Cap/Tab | 30 days | 50mg | 1170 | 1.1886 |
|  | Nifedipine 20mg ER Tab | 30 days | 30mg | 31 | 0.0315 |
|  | Prazosin 1mg Tab | 30 days | 5mg | 240 | 0.2438 |
|  | Propranolol 40mg Tab | 30 days | 0.16 g | 60.6 | 0.0616 |
| Hypothyroidism | Levothyroxine 50 mcg Cap/Tab | 30 days | 0.15mg | 585 | 0.5943 |
| Ischemic Heart Disease | Acetylsalicylic acid 75mg Cap/Tab | 30 days | 1 tablet | 99.9 | 0.1015 |
|  | Clopidogrel 75mg Cap/Tab | 30 days | 75mg | 484.8 | 0.4925 |
|  | Isosorbide Mononitrate 60 mg SR Tab | 30 days | 40mg | 266 | 0.2702 |
| Parkinson Disease | Levodopa + Carbidopa 275mg Cap/Tab | 30 days | 0.6g | 1881.8181 | 1.9118 |
| Peptic ulcer and gastro-oesophageal reflux disease (GORD) | Omeprazole 20mg Cap/Tab | 30 days | 20mg | 125.1 | 0.1271 |
|  | Ranitidine 150mg Cap/Tab | 30 days | 0.3g | 492 | 0.4998 |
| Psychosis | Haloperidol 1.5mg Cap/Tab | 30 days | 8mg | 60.8 | 0.0618 |
|  | Olanzapine 5mg Tab | 30 days | 10mg | 600 | 0.6096 |
|  | Risperidone 2mg Tab | 30 days | 5mg | 495 | 0.5029 |
|  | Lithium carbonate 250mg ER Tab | 30 days | 24mmol | 141.2806 | 0.1435 |
| Vitamins and minerals | Ferrous sulphate 200mg Cap/Tab | 30 days | 0.2g | 26.1 | 0.0265 |
|  | Folic Acid 1mg Cap/Tab | 30 days | 0.4mg | 21.96 | 0.0223 |
| Osteoporosis | Alendronate 70mg Cap/Tab | 30 days | 10mg | 15 | 0.0152 |
| **Acute Conditions** |  |  |  |  |  |
| Bacterial infection | Amoxicillin 125mg/5ml OS | 7 days | 250 mg TID | 73.5 | 0.0747 |
|  | Amoxicillin 250mg Cap/Tab | 7 days | 1.5g | 294 | 0.2987 |
|  | Amoxicillin+ Clavulanic acid 250mg Cap/Tab | 7 days | 1.5g | 158.4 | 0.1609 |
|  | Cefalexin 125mg/ml Suspension | 7 days | 2g | 141.28 | 0.1435 |
|  | Ceftriaxone injection 1 g/vial Powder for solution | 7 days | 2g | 4324 | 4.3928 |
|  | Cefuroxime axetil 250mg Cap/Tab | 7 days | 0.5g | 106 | 0.1077 |
|  | Ciprofloxacin 500mg Cap/Tab | 7 days | 1g | 18.44 | 0.0187 |
|  | Clarithromycin 250mg Cap/Tab | 7 days | 0.5g | 75 | 0.0762 |
|  | Cloxacillin 500mg Cap/Tab | 7 days | 2g | 21.988 | 0.0223 |
|  | Co-trimoxazole 40+200mg/5ml OS | 7 days | 240 mg BID | 11.4 | 0.0010 |
|  | Co-trimoxazole 400mg+80mg Cap/Tab | 7 days | 960mg BID | 56 | 0.0009 |
|  | Doxycycline 100mg Cap/Tab | 7 days | 0.1g | 9.3 | 0.0094 |
|  | Erythromycin 250mg Cap/Tab | 7 days | 1g | 28 | 0.0284 |
|  | Nalidixic acid 300mg/5ml Cap/Tab | 7 days | 12.5mg/kg (125mg) q6h | 53.7833 | 0.0546 |
|  | Nitrofurantoin 50mg Cap/Tab | 3 days | 50 mg/kg q6h | 10.56 | 0.0107 |
|  | Phenoxymethylpenicillin 250mg Tab | 7 days | 2g | 19.04 | 0.0193 |
| Constipation | Bisacodyl 5mg Suppository | 7 days | 10mg | 52 | 0.0528 |
|  | Lactulose 3.35g/5ml OS | 7 days | 6.7g | 0.95 | 0.0005 |
| Fungal infections | Mebendazole 500mg Cap/Tab | 7 days | 0.2g | 48 | 0.0488 |
|  | Nystatin 500000IU tab/cap | 7 days | 1.5 MU | 0.915 | 0.0009 |
| Herpes zoster | Aciclovir 200mg Cap/Tab | 7 days | 4g | 115 | 0.1168 |
| Ischemic Heart Disease | Glyceryl trinitrate 500 micrograms Tab | 7 days | 2.5mg | 40.5 | 0.0411 |
| Nematode infections | Diethylcarbamazine citrate 50mg Cap/Tab | 7 days | 0.4g | 16 | 0.0163 |
| Pain | Diclofenac 50 mg SR Cap/Tab | 7 days | 0.1g | 24 | 0.0244 |
|  | Ibuprofen 200mg Cap/Tab | 7 days | 1.2g | 6 | 0.0061 |
|  | Paracetamol 120mg/5ml OS | 7 days | 180 mg q6h | 76.5 | 0.0777 |
|  | Paracetamol 500mg Tab | 7 days | 3g | 14.7 | 0.0149 |
| Protozoal infections | Metronidazole 200mg Cap/Tab | 7 days | 2g | 36 | 0.0116 |
| Vertigo | Betahistine 8mg Cap/Tab | 7 days | 24mg | 29.025 | 0.0569 |
|  | Cetirizine 10mg Cap/Tab | 7 days | 10mg | 4.6 | 0.0010 |
| Vomiting | Domperidone 10mg Cap/Tab | 7 days | 30mg | 16.5 | 0.0488 |
|  | Metoclopramide 10mg Tab | 7 days | 30 mg | 1 | 0.0010 |
|  | Promethazine 25mg Tab | 7 days | 25mg | 0.54 | 0.0005 |

*Standard dose recommended for an adult or a child in 2–5-year age group weighing 10 kg as given in the British National Formulary (BNF) where DDD was not known

AIIRD- autoimmune inflammatory rheumatic diseases; BID – twice a day; DDD - defined daily dose; g- gram; mg – milligram;

ER; Extended release; Intravenous solution- IVS; LKR – Sri Lankan Rupees MDI; Metered Dose Inhaler; OS – Oral suspensions; PS- powder for solution; q6h – 6 hourly; SI – solution for injection; SR- Slow released; TID – three times a day; U – unit; tab- tablet; cap – capsule; UD – intrauterine device
